# Stratifying migraine patients based on dynamic pain provocation over the upper cervical spine

**DOI:** 10.1186/s10194-017-0808-0

**Published:** 2017-09-26

**Authors:** Kerstin Luedtke, Arne May

**Affiliations:** Department of Systems Neuroscience, University Medical Center Hamburg-Eppendorf, Martinistr. 52, 20246 Hamburg, Germany

**Keywords:** Neck pain, Musculoskeletal dysfunction, Headache, Upper cervical spine, Palpation, Neck muscles

## Abstract

**Background:**

Migraine patients usually report a high prevalence of neck pain preceding or during the migraine attack. A recent investigation of musculoskeletal dysfunctions in migraine patients concluded that neck pain is not simply a symptom of the migraine attack but corresponds to identifiable muscle and joint alterations. Particularly pain provocation using palpation of the joints in the upper cervical spine was significantly more prevalent in patients with migraine than in headache-free participants.

**Methods:**

One hundred seventy-nine migraineurs (diagnosed according to IHS classification criteria version III beta) and 73 age- and gender-matched healthy controls were examined by a physiotherapist blinded towards the diagnosis, using a palpation technique over the upper cervical spine. The palpation combined oscillating movements and sustained pressure.

**Findings:**

Using simple palpation of the upper cervical spine, migraine patients can be stratified into three groups: painfree (11%), local pain only (42%), and pain referred to the head during sustained pressure (47%). Combining both test components (palpation and sustained pressure) has a high sensitivity and specificity for migraine.

**Conclusions:**

The response to palpation of the upper cervical spine may indicate migraine subtypes. The presence of musculoskeletal dysfunctions of the upper cervical spine should be identified and treated to avoid ongoing nociceptive input into the trigeminocervical complex.

**Trial registration:**

German Clinical Trial Register DRKS-ID: DRKS00009622.

## Introduction

The influence of peripheral structures such as neck muscles or cervical joints on migraine and associated symptoms is the topic of an ongoing scientific debate. The generally accepted hypothesis, that migraine is mainly a disease of the central nervous system [1] is challenged by the high prevalence of neck pain reported by patients preceding or during the migraine attack [2, 3]. Migraineurs exhibit an increased frequency of musculoskeletal findings, [4–6], which could be based on a co-localization of cervical and trigeminal afferent input in the trigeminal nucleus [7], and a direct connection of peripheral nerves with the dura mater through cranial sutures as has been recently identified in animal models [8].

If there is a causal relationship between peripheral, i.e. cervical structures and migraine, there should be identifiable dysfunctions that are more common in patients with migraine than in headache-free participants. To investigate the frequency and the type of musculoskeletal findings in patients with migraine we recently published a Delphi survey reporting headache assessment tests (HATs) that reached international consensus amongst international experts in physical therapy and headaches, as the most useful for the evaluation of musculoskeletal dysfunctions in patients with headaches [9]. The HATs were subsequently applied to a sample of 138 migraine patients and 73 headache-free control participants using a blinded-examiner design [6]. Results of this study showed a surprisingly high prevalence of musculoskeletal (cervical and thoracic) findings in patients with migraine particularly for trigger points in the suboccipital muscles and for the manual examination of upper cervical joints. Ninety-three percent of the assessed patients had at least 3 different musculoskeletal dysfunctions [6].

These results are in line with publications on smaller migraine populations reporting higher numbers of trigger points [4, 10–12] and reduced upper cervical mobility during joint palpation [12] as well as referred pain during sustained pressure [13] compared to healthy controls. Other publications reported that only cervicogenic headache patients showed joint changes during palpation while migraine patients were not significantly different from healthy controls [14].

The aim of this short report is to evaluate the response of migraine patients compared to healthy controls to palpation of the upper cervical spine (with and without sustained pressure). We hypothesize that this easy procedure will clearly distinguish between patients and controls and will further allow the definintion of clinical subtypes. Potential implications for the underlying pathophysiology and for the clinical management are discussed.

## Methods

### Participants

The data presented here are based on 252 participants (179 migraineurs and 73 age- and gender-matched healthy controls). Procedures are described in detail elsewhere [6]. In summary, one physiotherapist (more than 20 years postgraduate experience), blinded towards the diagnosis, examined all participants following the recommendations of the international headache experts [9]. Patients were included if they were adult, had a minimum of 2 years of migraine, diagnosed by an experienced neurologist and expert in the diagnosis and treatment of headaches accoring to the IHS classification system [1], and had a minimum of 6 attacks per year. Age- and gender-matched control participants had a maximum of 2 headache episodes per year that did not fulfill the criteria for any primary headache other than episodic tension-type headache. Patients and controls were excluded if they had any pathology in the neck, including diagnosed osteoarthritis, rheumatoid or psychiatric disease or craniomandibular dysfunction. Patients diagnosed with episodic migraine had to be headache-free on the day of the appointment and during the 48 h prior to the examination. Chronic migraine patients were required to be migraine-free on the day of the appointment.

### Procedures

**Fig. 1:**
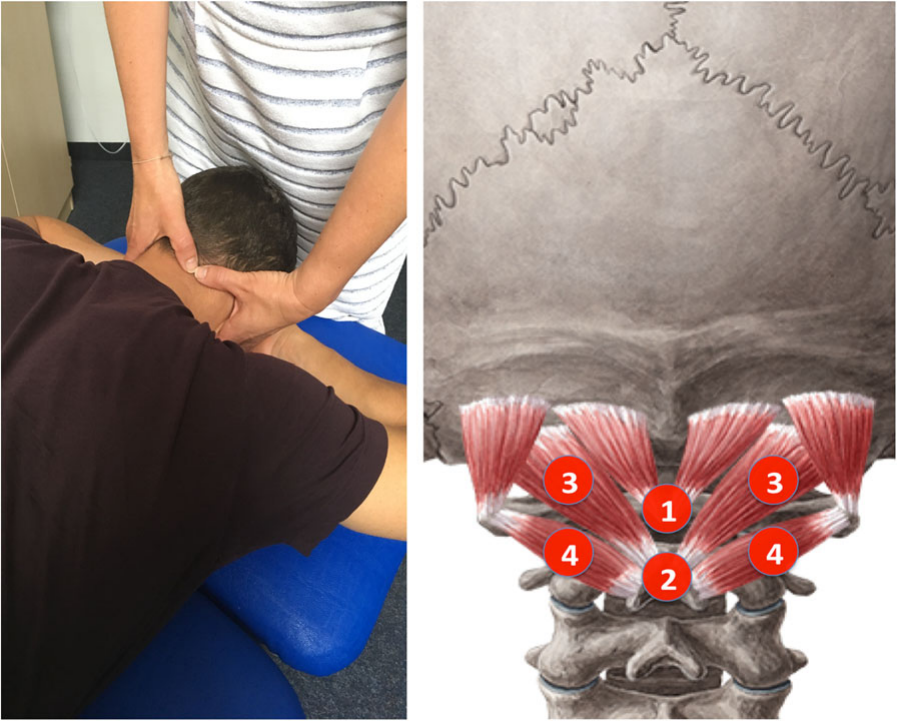
Palpation of the upper cervical spine. 1 = midline C1; 2 = spinuous process C2; 3 = atlantoocipital joint; 4 = antlantoaxial joint

Palpation of the upper cervical spine was conducted with the patient positioned in prone, with his/her hands supporting the head. Manual pressure was applied over the midline of C1 and the spinuous processes of C2 and C3. Subsequently pressure was applied over the area of the joint C0/1 and C1/2 on the right and on the left hand side. The exact testing procedure was described by Maitland [15] to test joint dysfunction and by Watson & Drummond to examine headache patients with a sustained pressure technique [13]. Outcomes were pain provocation (yes/no) and referred pain to the head during sustained (maximum of 5 s) pressure (yes/no) (see Fig. 1).

### Data analysis

To distinguish subgroups of migraineurs responding differently to palpation of the upper cervical spine, the analysis focused on the number and percentage of patients, who reported either local pain provocation during the manual examination and/or pain referred to the head during sustained pressure. Results across groups were tested for statistical significance using the Chi^2^ Test.

Test results were evaluated for a correlation with age using biserial point correlation to accommodate for undiagnosed age-related changes. Pearson’s corrlelation was also calculated for the number of headache days per month. A further correlation was conducted with the reported side of the headache symptoms using Cramer’s V for nominal data. The sensitivity and specificity of both components of the test as well as the positive and negative predictive values were calculated using cross-tabulation. All analyses were conducted in SPSS Version 24 (IBM).

## Findings

**Fig. 2:**
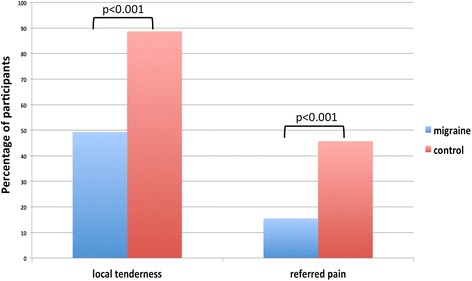
Percentages of local and referred pain for migraine patients and controls

One hundred ninety-five of the 252 participants (descriptive data in Table 1) showed local tenderness on palpation of the upper cervical spine, 159 of these were migraine patients (Fig. 2). Ninety-three participants (82 migraine patients) reported referred pain to the head during sustained pressure (Fig. 2).

**Table 1:** Sample description

	Migraineurs (*n* = 179)	Controls (*n* = 73)
Age mean (SD)	40 (15)	40 (13)
Females %	92%	84%
Headache days per month mean (SD)	12 (8)	N/A
Disease duration years(SD)	19 (13)	N/A

Based on these numbers, local pain provocation during palpation of the upper cervical spine showed a high sensitivity of 0.888 and a lower specificity of 0.507, because half of the non-headache participants also reported local pain. The positive predictive value was 0.813 the negative predictive value 0.649.

In contrast, pain referral to the head during sustained pressure showed a sensitivity of 0.466 and a high specificity of 0.845. The corresponding positive predictive value was 0.879 and the negative predictive value was 0.387 (Table 2).

**Table 2:** Contingency table for local pain provocation and referred pain in migraine patients and control

		No pain	Local pain	Total	No referred pain	Referred pain	Total
Control	Count	37	36	73	60	11	71
	%	50,7%	49,3%	100,0%	84,5%	15,5%	100,0%
Migraine	Count	20	159	179	94	82	176
	%	11,2%	88,8%	100,0%	53,4%	46,6%	100,0%
Total	Count	57	195	252	154	93	247
	%	22,6%	77,4%	100,0%	62,3%	37,7%	100,0%

There was no significant correlation of the test result with age for local pain provocation (*p* = 0.513) or for referral to the head (*p* = 0.846) but a significant correlation with headache days per month (*p* = 0.027 for pain provocation and *p* = 0.019 for referred pain).

Cramer’s V to correlate the typical symptomatic headache side (left, right or both) with the side of the pain provocation (left, right, both) was not significant with *p* = 0.620.

## Discussion

Pain provocation during palpation of the upper cervical spine was significantly more present in migraine patients than in healthy controls. Pain during palpation and sustained pressure classified migraine patients into 3 subtypes:

1. Patients who did not report any pain during palpation,
2. Patients who reported only local pain,
3. Patients who additionally reported referred pain to the head during sustained pressure.

Approximately 80% of the migraine patients reported local tenderness during palpation; half of these also had referred pain to the head. The sensitivity and specificity for the two tests in combination is high. These findings confirm the result of a previous smaller scale study on head pain referral in patients with tension-type headache and migraine reporting that 14/14 patients with tension-type headache and 19/20 patients with migraine showed the same phenomenon of head referral during sustained pressure over the upper cervical spine [13]. An earlier study, using a range of physical examination procedures in participants with migraine, tension-type headache and cervicogenic headache, did not identify upper cervical joint dysfunctions in the (albeit small) proportion of migraine patients and the authors concluded that cervical joint findings are part of the diagnostic procedure to distinguish cervicogenic from primary headaches [14]. In this larger sample, both subtypes (with and without tenderness in the neck) were found. This raises the question whether neck tenderness is an indicator for distinct subtypes of migraine: patients with neck pain as a simple symptom prior or during the attack without any identifiable changes in the periphery, and patients where the neck plays a crucial role either as a trigger or as a perpetuator for migraine attacks. The second group, if untreated, could be susceptible to ongoing nociceptive input from the neck, which activates the trigeminocervical system and subsequently leads to more frequent headache attacks, eventually contributing to the transition from episodic to chronic headache [16, 17]. Although these statements were originally related to tension-type headache, considering the partially related pathobiology, this might also be true for migraine.

Manual examination of the C0/1 and the C1/2 joint regions on either side, requires pressure through the suboccipital muscles rectus capitis posterior major (and possibly minor) and obliquus capitis inferior (and partially superior), hence pain provocation during palpation could either indicate muscle or joint changes. While joint changes have not been investigated in migraine patients, upper cervical MRI changes in patients with cervicogenic headaches could not be confirmed [18]. Muscle changes, such as trigger points, however, have been reported frequently in the past [4, 10, 12]. Suboccipital muscles are supplied by the dorsal rami of the first and second spinal nerves. The second spinal nerve is also the source of the greater and lesser occipital nerves. The close proximity of these sensitive structures to the upper cervical joints as well as the convergence of afferent fibers of the cervical and the trigeminal system within the trigeminal nucleus, provide mechanical explanations for the head pain referral during sustained pressure but also for local tenderness during palpation. Consequenty, injections or stimulation of the greater occipital nerve (GON) have been shown to be effective treatment options for some patients with chronic migraine [19, 20]. The mechanism explaining why an intervention to the GON should eliminate trigeminal symptoms is not fully understood but might at least partially rely on the same trigeminocervical convergence theory. One of the clinical predictors for the effectiveness of a greater occipital nerve block is local tenderness over the area of the GON [21], hence both techniques, palpation of the GON and manual joint examination might identify the same subpopulations of migraine patients and might therefore be useful for the choice of the most promising treatment approach.

The sensitivity to palpation in our patients was not correlated to the dominant side of headache. This could be explained by the fact that only approximately 15% of our participants suffered from side-locked headache, while the majority had migraine that could change sides during or across attacks. The obvious non-mechanical explanation for bilateral pain provocation is, that local tenderness and referred pain indicate a generalised hypersensitivity of the suboccipital region due to central sensitisation in this particular subgroup of migraine patients [22]. However, most of the previous publications on hypersensitivity or allodynia in migraine patients, if measured interictally, reported negative or conflicting results. The currently agreed opinion is therefore, that there is probably no difference between migraineurs and controls interictally and that allodynia is part of the attack in a subgroup of patients [23]. Kitaj et al., however, reported lowered pain thresholds in chronic migraine patients compared to episodic migraine patients, even when measured interictally, indicating that disease duration could contribute to the development of central sensitisation. This was particularly evident in the neck region [24].

Whether peripheral tissue such as joints or muscles can indeed trigger migraine attacks and/or contribute to the transition to chronic migraine remains to be investigated. If this hypothesis is confirmed, it is essential to detect and treat musculoskeletal dysfunctions appropriately to reduce incoming nociceptive signals. It is therefore proposed to include manual joint examination into the routine physical examination procedure of migraine patients and to use both components (palpation and sustained pressure) of the tests in combination to reach a high specificity and sensitivity.
